# Injury-Related Deaths according to Environmental, Demographic, and Lifestyle Factors

**DOI:** 10.1155/2019/6942787

**Published:** 2019-03-03

**Authors:** Ray M. Merrill

**Affiliations:** Department of Public Health, College of Life Sciences, Brigham Young University, Provo, UT, USA

## Abstract

**Background:**

Environmental, demographic, and lifestyle variables have been associated with injury-related deaths. The current study identifies the simultaneous association of selected environmental, demographic, and lifestyle variables with deaths from homicide, unintentional injuries, and suicide.

**Materials and Methods:**

Analyses are based on county-level mortality data in the contiguous United States, 2011–15. Basic summary statistics and Poisson regression were used to evaluate the data.

**Results:**

The selected causes of death were impacted differently by age, sex, and race: for homicide, mortality rates were greater in ages 20–39, males, and blacks; for unintentional injuries, the rates increased with age, most noticeably in the oldest age group, and were highest among males and whites; and for suicide, the rates tended to increase with age and were greater in males and whites. Mortality rates from homicide were positively associated with poverty, cigarette smoking, air temperature, and leisure-time physical inactivity. They were negatively associated with precipitation and sunlight. Mortality rates from unintentional injuries were positively associated with altitude, cigarette smoking, air temperature, poverty, obesity, and precipitation. They were negatively associated with population density. Mortality rates from suicides were positively associated with altitude, cigarette smoking, obesity, air temperature, and precipitation and negatively associated with population density.

**Conclusion:**

The results confirm and extend previous research in which death from homicide, unintentional injuries, and suicide are distinctly associated with a combination of environmental, demographic, and lifestyle variables. The findings may be useful in developing strategies for reducing injury-related deaths.

## 1. Introduction

Injury-related situations explain most deaths in the age range 1–44 in the United States [1]. In 2015, deaths from unintentional injuries occurred 3.3 times more often than death from suicides and death from suicides occurred 2.5 times more often than death from homicides. Specifically, the number of deaths from unintentional injuries (e.g., motor vehicles, poisoning, fire, falling, suffocation, and drowning) was 146,571 (45.6 per 100,000), from suicides was 44,193 (13.7 per 100,000), and from homicides was 17,793 (5.5 per 100,000) [2].

Environmental factors such as ambient air temperature, altitude, and precipitation have been associated with injury-related deaths. For example, a recent study showed that increases in ambient air temperature explained 10% of the variance in violent crime in Finland [3]. The study further suggested that the effect of ambient air temperature on violent crime is mediated by the serotonergic system. Specifically, higher ambient air temperature is associated with changes in serotonin transporter density that, in turn, increases impulsivity and irritability. Other studies have linked increasing ambient air temperature with higher violent suicide rates, violence, and trauma [4, 5]. There is also evidence of greater risk-taking behavior with increased heat exposure [6]. Violent crime and risk-taking behavior may result in death from unintentional injury (e.g., motor vehicle crashes and accidental firearm discharge) or homicide (death inflicted by another person with the intent to harm).

Several studies have found a positive relationship between altitude and suicide rates, after adjusting for demographic characteristics, gun ownership, and population density [7–13]. Increase in altitude is associated with an exponential decrease in atmospheric pressure [14]. As atmospheric pressure decreases, the partial pressure of inspired oxygen falls and less oxygen is absorbed into the body, thus causing hypoxia [15]. Metabolic stress from hypoxia negatively affects mood and increases the risk of depressive symptoms, thereby increasing the risk of suicide [8–11, 16–22].

Adverse effects of higher altitude on psychological mood (irritability, hostility, and depression) and cognitive function (mental skills, reaction time, psychomotor performance, memory, etc.) are well described [23–26]. Hypoxic conditions explain the impairment in mood and cognition that occurs with higher altitude exposure [20, 27]. Hypoxia associated with higher altitude has been associated with greater occupational injuries among miners [28] and higher rates of errors [29, 30]. Psychological states such as depression have been associated with increased risk of subsequent unintentional injury [31, 32].

Rainfall and extreme precipitation events have been shown to increase the risk of automobile accidents [33–35], through loss of contact between the tire and the road, impaired visibility, and strain on cognitive capacity [36, 37]. Weather conditions explained 22% of car accidents during 2005–14 [38]. Of those involved in these accidents, about 1.3% were killed. Precipitation can also lead to injuries and deaths from falls, drowning, and hypothermia.

Along with environmental factors, injury-related deaths may be influenced by poverty, smoking, obesity, and other demographic and lifestyle variables. For example, research has associated higher poverty with greater levels of homicide [39], suicide [40, 41], and deaths from unintentional injuries [42, 43]; cigarette smoking with suicide [44–46] and deaths from unintentional injuries [47]; and obesity with suicide [48] and death from motor vehicle collision [49]. However, a recent systematic review and meta-analysis showed an inverse association between obesity and suicide [50].

The relative contribution of environmental, demographic, and lifestyle variables on injury-related deaths has not been previously assessed. The purpose of the current study was to identify the association between injury-related deaths and selected environmental, demographic, and lifestyle variables and injury-related deaths. The association between the environmental, demographic, and lifestyle variables will also be considered.

## 2. Materials and Methods

Analyses were based on county-level mortality data for homicide and legal intervention (homicide), unintentional injuries, and suicide in the contiguous United States [51]. Federal Information Processing Standard (FIPS) codes identify counties in the United States. Mortality groupings are based on the International Classification of Diseases (ICD). Mortality data cover 2011 through 2015. Other variables included in this study, measured on the county level, were average daily sunlight (kJ/m^2^), altitude (m), average maximum daily temperature (F), average fine particulate matter (*µ*g/m³), average daily precipitation (mm), percent living in poverty, percent of adults who smoke cigarettes, percent urban residents, percent obese (body mass index ≥ 30), and percent leisure-time physical inactivity. The number of counties with available information on these environmental, demographic, and lifestyle variables ranged from 3108 to 3142, as described in Table 1.

Average daily sunlight (kJ/m^2^), daily air temperature (F), and daily precipitation (mm) values are from the North America Land Data Assimilation System through the CDC Wonder database, 2007–11 [52]. Fine particulate matter (*µ*g/m³) values are from the CDC wonder database, 2007–11 [52]. Altitude (meters) values are from the Geographic Names Information System from the United States Geological Survey [53].

Poverty values for 2013 are from the United States Census Bureau [54]. This measure is based on estimates of the level of income required to cover basic needs. Poverty involves those people residing in households with income below what is needed to cover basic needs. Cigarette-smoking prevalence estimates (age-adjusted 2000 US standard population) in adults, 1996–2012, are taken from a previous report [55]. The percent of the population living in urban areas was obtained from 2010 individual state reports from the 2010 Census of Population and Housing [56]. The population density per square mile of land area for 2010 is from the United States Census Bureau [57]. Prevalence values for obesity and leisure-time physical inactivity (age-adjusted 2000 US standard population) for 2013 were obtained from the Centers for Disease Control and Prevention [58].

The environmental, demographic, and lifestyle variables considered in this study were described using summary statistical values (mean, median, standard deviation, minimum, and maximum) across the counties. Poisson regression was used to assess the association between the mortality rates from homicide, unintentional injuries, and suicide with the environmental, demographic, and lifestyle variables. Variables were added to the models, one at a time, and dropped if they were not statistically significant at the 0.05 level (or close to unity but significant merely because of large numbers). Age, sex, and race were retained in each model. Resulting mortality rate ratios were adjusted for age, sex, race, and other variables that significantly contributed to the model. Statistical analyses were performed using SAS 9.4 (SAS Institute, Cary, NC, USA, 2012).

## 3. Results

Relative mortality rates of homicide, unintentional injuries, and suicide in the United States are presented according to age, sex, and race for the years 2011–15 in Table 2. Age, sex, and race impacted these causes of death differently. For homicide, the rates were greater in ages 20–39, males, and blacks. There was no difference in rates between people in the youngest and oldest age groups. Other racial groups (Asian/Pacific Islander and American Indian/Alaska Native) had the lowest rates. For unintentional injuries, the rates increased with age, most noticeably in the oldest age group, and were highest among males and whites. Rates were similar across ages 20 through 79 and lowest among those in the other racial group. For suicide, the rates were greater in ages 20 years and older, males, and whites. Rates were similar across ages 20–79 and between blacks and those in the other racial group.

Summary statistics for selected county-specific environmental, demographic, and lifestyle variables are shown in Table 1. The variables are normally distributed, with the exceptions of altitude and population density, which are positively skewed. Each variable shows large variation across the counties.

Relative mortality rates of homicide in the United States are presented according to selected environmental, demographic, and lifestyle variables for 2011–15 in Table 3. Relative mortality rates from homicide are adjusted for age, sex, and race or fully adjusted for the variables with results appearing in the table. Counties with higher average daily temperature, poverty, adult smoking, and physical inactivity have higher mortality rates from homicide. Counties with higher average daily sunlight, average daily precipitation, and population density have lower mortality rates from homicide. In general, the lowest rates of death from homicide are in counties with higher average daily sunlight, lower average daily air temperature (bottom 25%), higher average daily precipitation, lower poverty (bottom 25%), lower adult smoking (bottom 25%), higher population density, and lower leisure-time physical inactivity (bottom 25%).

Relative mortality rates of unintentional injuries in the United States are presented according to selected environmental, demographic, and lifestyle variables for the time period 2011–15 in Table 4. Counties with higher altitude, average daily temperature, average daily precipitation, poverty, adult smoking, and obesity have higher mortality rates from unintentional injuries. Counties with higher population density have lower mortality rates from unintentional injuries. In general, the lowest rates of death from unintentional injuries are in counties with lower altitude (bottom 25%), lower average daily air temperature (bottom 75%), lower average daily precipitation (bottom 25%), lower poverty (bottom 75%), lower adult smoking (bottom 25%), higher population density (top 75%), and lower obesity (bottom 25%).

Relative mortality rates of suicide in the United States are presented according to selected environmental, demographic, and lifestyle variables for the time period 2011–15 in Table 5. Counties with higher altitude, average maximum daily temperature, average daily precipitation, adult smokers, and obesity have higher mortality rates from suicide. Counties with higher population density have lower mortality rates from suicide. In general, the lowest rates of death from suicide are in counties with low altitude (bottom 25%), low average maximum daily air temperature (bottom 50%), lower average daily precipitation (bottom 25%), low adult smoking (bottom 25%), higher poverty (top 50%), higher population density (top 75%), and lower obesity (bottom 25%).

Average fine particulate matter above the median was significantly associated with higher levels of homicide, accidents, and suicide after adjusting for age, sex, and race. However, with the addition of adult smoking, average fine particulate matter became insignificant in each of the models. Higher urban residency was significantly associated with greater levels of homicide, accidents, and suicide after adjusting for age, sex, and race. Yet, when population density per square mile of land was also added to the models, urban residency became insignificant. Finally, strong correlation between obesity and physical inactivity resulted in the dominant of the two variables being retained in each of the models.

## 4. Discussion

This study explored the association between injury-related mortality rates and selected environmental, demographic, and lifestyle variables in the United States. Mortality rates from homicide, unintentional injuries, and suicide according to age, sex, and race are consistent with a report from the National Center for Health Statistics, 2016 [59]. Each of these causes of death was simultaneously associated with a combination of environmental, demographic, and lifestyle variables.

Deaths rates from homicide were greater in counties with higher average daily temperature, poverty, adult smoking, and physical inactivity. Rates were lower in areas with higher average daily sunlight, average daily precipitation, and population per square mile. Other studies have associated homicide with higher temperature and poverty [3, 5, 6, 39]. The result on population density is unclear. It does not appear that other research has assessed the association between homicide and smoking, precipitation, and sunlight. It is not known whether the associations in the current study are explained by other covariates, not included in our study. Higher temperature may be associated with more homicide deaths because greater temperature causes people to spend more time outside their homes [60]. In a similar manner, we found that more precipitation was related to lower homicide mortality rates. The observed positive association between leisure-time physical inactivity and homicide may be because people in places with greater violent crime are less inclined to go outdoors to exercise. One study found that adult walking was deterred in areas with higher violent crime [61]. Greater sunlight exposure is associated with higher levels of serotonin, which correlates with better mood, lower anxiety, and depression [62], and possibly lower risk-taking behavior [63]. Sunlight is also a source of vitamin D. Deficiency of vitamin D has been associated with mood disorders and depression [64, 65]. Research has identified increased risk of violent crime among individuals with mood disorders, anxiety, or depression [66–68].

Mortality rates from unintentional injuries are higher in counties with higher altitude, average daily temperature, average daily precipitation, poverty, adult smoking, and obesity. There is an inverse association between mortality rates from unintentional injuries and population density. The association with altitude may be because higher altitude is associated with places where deaths are more likely to occur from injuries (e.g., falls from mountain climbing or trauma from ski crashes). Acute mountain sickness, which occurs when the body does not get enough oxygen [15], has symptoms like dizziness and muscle aches that can increase unintentional injuries.

Cigarette smoking has been identified as a leading cause of fire disaster and resulting death, fatal automobile accidents, and injuries at work and other unintentional injuries [36, 69, 70]. Reasons for the association between smoking and injuries may be because of distraction, direct toxicity, and confounding factors (smoking-related medical conditions, personality, and behavior characteristics) [47].

The association between air temperature and unintentional injuries is consistent with other research showing that, at hotter temperatures, individuals perceive the same risky behavior as less risky and participate in more risk-taking behavior [6].

The association between higher poverty and death from unintentional injuries is consistent with other research [42, 43]; the association between obesity and death from unintentional injuries is consistent with previous research [49], and the positive association between precipitation and unintentional injuries is consistent with other research [33–38]. Other research has found that urban settings have lower levels of death from unintentional injuries, possibly because rural areas have higher levels of motor vehicle trauma, and exposure to hazardous firearms, farm machinery, falls, poisoning, and open water [71, 72]. The relative lack of emergency care and emergency care resources in rural areas may further explain higher deaths from unintentional injuries in lower population density areas [73].

Mortality rates from suicide are higher in counties with higher altitude, average maximum daily temperature, average daily precipitation, adult smokers, and obesity have higher mortality rates from suicide. The observed association between suicide and altitude is consistent with previous research [7–13]. The mechanism may involve hypoxia [15], wherein hypoxia increases the risk of depressive symptoms and risk of suicide [8–11, 16–22]. The observed association between suicide and smoking has also been observed previously [44–46]. The positive association observed between suicide and obesity was seen in another recent study [48] but not identified in a review study [50]. Consistent with the current finding, higher ambient air temperature has been previously associated with greater violent suicide rates [4]. It is not clear why precipitation was positively related to suicide. It may be that greater precipitation limits sun synthesized vitamin D, increases feelings of isolation, and adversely affects other feelings and possible behaviors associated with suicide. The negative association observed between suicide and population density is consistent with other research [74].

Average fine particulate matter was positively associated with suicide, after adjusting for age, sex, and race. This result is consistent with a study finding a positive association between exposure to nitrogen dioxide, particulate matter, and sulfur dioxide and suicide [75]. However, we found that further adjustment for other variables resulted in no association between air pollution and suicide, as consistent with another study adjusting for potential confounders [76].

The primary limitation of this study is that the measures are on the county level rather than the individual level. Thus, ecologic fallacy may play a role in our assessment of associations. Nevertheless, the environmental variables are similarly experienced among people within each county, for the most part. The large sample size tended to produce statistically significant results that may not be of practical importance. The environmental variables considered are largely not modifiable and may be less clinically useful for physicians and public health officials. Although the study focuses on the influence of environmental variables, a limited number of other variables were considered. This list does not include all variables known to influence injury (e.g., alcohol use). Finally, because the data are not longitudinal, drawing conclusions about causal directions is problematic (e.g., between obesity and smoking and smoking and poverty). Yet, for some of the variables, the causal direction is clear (e.g., higher temperature leads to more leisure-time physical activity, obesity, and poverty).

## 5. Conclusion

The findings in this study show the relative contribution of environmental, demographic, and lifestyle variables on injury-related deaths. Several databases were combined to provide a clearer understanding of factors associated with injury-related deaths. Mortality rates from homicide are positively associated with poverty, cigarette smoking, air temperature, and leisure-time physical inactivity and negatively associated with precipitation and sunlight; mortality rates from unintentional injuries are positively associated with altitude, cigarette smoking, air temperature, poverty, obesity, and precipitation and negatively associated with population density; and suicides are positively associated with altitude, cigarette smoking, obesity, air temperature, and precipitation and negatively associated with population density. Future study may determine more clearly the impact of the environmental variables assessed in this study on mortality rates from homicide, unintentional injuries, and suicide by considering seasonality.

## Figures and Tables

**Table 1:** Selected county-specific environmental, demographic, and lifestyle variables in the contiguous United States.

	Number	Mean	SD	Median	Minimum	Maximum
*Environmental*						
Average daily sunlight (kJ/m^2^)	3111	16400.0	1605.0	16106.0	12689.0	21191.0
Altitude (m)	3108	424.8	494.4	269.0	−17.0	3590.0
Average daily maximum air temperature (F)	3111	65.4	9.3	64.8	38.4	87.5
Average fine particulate matter (*μ*g/m^3^)	3111	11.7	1.4	11.9	7.9	14.3
Average daily precipitation (mm)	3111	2.7	0.9	3.0	0.2	7.1

*Demographic/lifestyle*						
% in poverty, 2013	3109	17.3	6.6	16.4	3.0	55.1
% adult smokers	3108	26.0	3.8	26.1	9.3	40.3
% urban, 2010 census	3109	43.3	31.9	42.7	0.0	100.0
Population density per square mile of land area, 2010	3109	261.5	1733.2	46.0	0	69468.0
% obese, age-adjusted, 2013	3142	30.8	4.6	31.0	11.8	47.9
% leisure-time physical inactivity, age-adjusted, 2013	3142	24.7	5.0	24.5	8.4	39.8

**Table 2:** Relative mortality rates from homicide and legal intervention, accidents and adverse effects, and suicide and self-inflicted injury in the contiguous United States according to age, sex, and race, 2011–15.

	Homicide and legal intervention					Accidents and adverse events					Suicide and self-inflicted injury				
	Number	Relative rate	95% LCL	95% UCL	Pr > ChiSq	Number	Relative rate	95% LCL	95% UCL	Pr > ChiSq	Number	Relative rate	95% LCL	95% UCL	Pr > ChiSq
*Age*															
0–19	9387	1.00				32703	1.00				6481	1.00			
20–39	41537	3.35	3.26	3.44	<0.0001	162261	4.56	4.50	4.63	<0.0001	58157	6.07	5.86	6.28	<0.0001
40–59	15886	1.59	1.54	1.64	<0.0001	192725	5.34	5.27	5.42	<0.0001	77496	8.07	7.80	8.35	<0.0001
60–79	2749	0.88	0.83	0.93	<0.0001	122681	5.65	5.57	5.74	<0.0001	33689	6.90	6.66	7.14	<0.0001
≥80	134	1.18	0.80	1.75	0.4077	143786	31.41	30.96	31.87	<0.0001	6317	10.71	10.26	11.18	<0.0001

*Sex*															
Female	12648	1.00				249700	1.00				40859	1.00			
Male	63852	5.12	4.98	5.27	<0.0001	426206	1.94	1.93	1.95	<0.0001	160245	3.30	3.26	3.35	<0.0001

*Race*															
White	34811	1.00				586933	1.00				187609	1.00			
Black	40436	6.53	6.41	6.65	<0.0001	67655	0.86	0.85	0.87	<0.0001	9550	0.48	0.47	0.49	<0.0001
Other	1717	0.76	0.70	0.82	<0.0001	18425	0.46	0.45	0.47	<0.0001	5942	0.48	0.47	0.50	<0.0001
	81194					679522					208287				

*Note.* Homicide consists of deaths from injuries inflicted by another person with the intention of harming. Legal intervention consists of deaths from injuries inflicted by law enforcement agents. In addition, for each model, the relative rates were simultaneously estimated across the three variables.

**Table 3:** Relative mortality rates of death from homicide and legal intervention according to selected environmental, demographic, and lifestyle variables in the contiguous United States, 2011–15.

Quartile groupings	Adjusted for age, sex, and race				Adjusted for age, sex, race, and the other variables with results listed			
	Relative rate	95% LCL	95% UCL	Pr > ChiSq	Relative rate	95% LCL	95% UCL	Pr > ChiSq
*Average daily sunlight (kJ/m* ^*2*^)								
<15050	1.00				1.00			
15050–16105	1.02	0.99	1.05	0.1137	1.01	0.97	1.04	0.7064
16106–17720	0.70	0.68	0.72	<0.0001	0.69	0.66	0.72	<0.0001
≥17721	0.95	0.93	0.98	0.0001	0.80	0.75	0.84	<0.0001

*Altitude (m)* ^*∗*^								
<268	1.00							
269–476	0.99	0.97	1.02	0.4816				
477–1040	0.95	0.92	0.98	0.0004				
≥1041	1.08	1.02	1.13	0.0051				

*Average maximum daily temperature (F)*								
<58.1	1.00				1.00			
58.1–64.7	1.03	1.00	1.06	0.0725	1.07	1.03	1.11	0.0003
64.8–73.0	1.03	1.00	1.07	0.0304	1.30	1.24	1.36	<0.0001
≥73.1	1.09	1.06	1.12	<0.0001	1.28	1.21	1.35	<0.0001

*Average fine particulate matter (µg/m* ^*3*^)								
<10.5	1.00							
10.5–11.8	0.91	0.89	0.93	<0.0001				
11.9–12.8	1.14	1.12	1.17	<0.0001				
≥12.9	1.12	1.09	1.15	<0.0001				

*Average daily precipitation (mm)*								
<2.08	1.00				1.00			
2.08–2.97	0.93	0.91	0.96	<0.0001	0.75	0.72	0.78	<0.0001
2.98–3.37	0.95	0.92	0.97	<0.0001	0.78	0.76	0.81	<0.0001
≥3.38	0.84	0.82	0.86	<0.0001	0.69	0.66	0.71	<0.0001

*% in poverty, 2013*								
3.0–12.5	1.00				1.00			
12.6–16.3	1.30	1.26	1.35	<0.0001	1.19	1.15	1.24	<0.0001
16.4–20.8	1.79	1.74	1.85	<0.0001	1.52	1.47	1.57	<0.0001
20.9–55.1	2.14	2.07	2.21	<0.0001	1.77	1.69	1.84	<0.0001

*% adult smokers*								
9.3–23.6	1.00				1.00			
23.6–26.1	1.42	1.39	1.45	<0.0001	1.39	1.35	1.43	<0.0001
26.2–28.5	1.40	1.36	1.44	<0.0001	1.30	1.25	1.35	<0.0001
28.6–40.3	2.06	2.01	2.12	<0.0001	1.63	1.56	1.70	<0.0001

*% urban, 2010 census*								
0–17.3	1.00							
17.3–43.7	1.47	1.32	1.65	<0.0001				
43.8–69.1	0.86	0.79	0.94	0.0013				
69.2–100	0.63	0.59	0.67	<0.0001				

*Population density per square mile of land area, 2010* ^*∗*^								
0–45	1.00				1.00			
46–114	0.54	0.50	0.60	<0.0001	0.63	0.57	0.70	<0.0001
115–379	0.42	0.38	0.45	<0.0001	0.55	0.50	0.60	<0.0001
380–69468	0.39	0.36	0.42	<0.0001	0.59	0.54	0.64	<0.0001

*% obese, age-adjusted, 2013*								
11.8–28.0	1.00							
28.0–30.9	1.19	1.16	1.22	<0.0001				
31.0–33.7	1.59	1.56	1.63	<0.0001				
33.8–47.9	1.43	1.38	1.49	<0.0001				

*% leisure-time physical inactivity*, *age-adjusted*, *2013*								
9.7–21.4	1.00				1.00			
21.4–24.4	1.12	1.09	1.14	<0.0001	1.09	1.06	1.12	<0.0001
24.5–28.0	1.31	1.28	1.34	<0.0001	1.08	1.05	1.12	<0.0001
28.0–39.8	1.25	1.20	1.29	<0.0001	1.10	1.05	1.16	0.0001

*Note.* Homicide consists of deaths from injuries inflicted by another person with the intention of harming. Legal intervention consists of deaths from injuries inflicted by law enforcement agents. ^*∗*^Altitude and population density groupings represent 50%, 25%, 15%, and 10% of the counties.

**Table 4:** Relative mortality rates of death from accidents and adverse events according to selected environmental, demographic, and lifestyle variables in the contiguous United States, 2011–15.

Quartile groupings	Adjusted for age, sex, and race				Adjusted for age, sex, race, and the other variables with results listed			
	Relative rate	95% LCL	95% UCL	Pr > ChiSq	Relative rate	95% LCL	95% UCL	Pr > ChiSq
*Average daily sunlight (kJ/m* ^*2*^)								
<15050	1.00							
15050–16105	1.05	1.04	1.06	<0.0001				
16106–17720	1.05	1.04	1.06	<0.0001				
≥17721	0.96	0.96	0.97	<0.0001				

*Altitude (m)* ^*∗*^								
<268	1.00				1.00			
269–476	1.09	1.08	1.09	<0.0001	1.08	1.08	1.09	<0.0001
477–1040	0.91	0.90	0.92	<0.0001	1.01	1.00	1.02	0.0924
≥1041	1.23	1.22	1.25	<0.0001	1.37	1.35	1.39	<0.0001

*Average maximum daily temperature (F)*								
<58.1	1.00				1.00			
58.1–64.7	0.95	0.95	0.96	<0.0001	0.98	0.97	0.99	<0.0001
64.8–73.0	0.97	0.96	0.98	<0.0001	1.01	1.00	1.01	0.2145
≥73.1	1.03	1.03	1.04	<0.0001	1.09	1.08	1.10	<0.0001

*Average fine particulate matter (µg/m* ^*3*^)								
<10.5	1.00							
10.5–11.8	1.00	1.00	1.01	0.2718				
11.9–12.8	1.13	1.12	1.14	<0.0001				
≥12.9	1.19	1.18	1.20	<0.0001				

*Average daily precipitation (mm)*								
<2.08	1.00				1.00			
2.08–2.97	1.14	1.13	1.15	<0.0001	1.05	1.04	1.06	<0.0001
2.98–3.37	1.10	1.09	1.11	<0.0001	1.05	1.04	1.06	<0.0001
≥3.38	1.12	1.12	1.13	<0.0001	1.10	1.09	1.11	<0.0001

*% in poverty, 2013*								
3.0–12.5	1.00				1.00			
12.6–16.3	1.13	1.12	1.14	<0.0001	1.00	0.99	1.01	0.6699
16.4–20.8	1.14	1.13	1.14	<0.0001	0.99	0.98	1.00	0.0304
20.9–55.1	1.31	1.29	1.32	<0.0001	1.05	1.04	1.06	<0.0001

*% adult smokers*								
9.3–23.6	1.00				1.00			
23.6–26.1	1.29	1.28	1.29	<0.0001	1.18	1.17	1.19	<0.0001
26.2–28.5	1.44	1.43	1.45	<0.0001	1.26	1.25	1.27	<0.0001
28.6–40.3	1.69	1.67	1.70	<0.0001	1.37	1.36	1.39	<0.0001

*% urban, 2010 census*								
0–17.3	1.00							
17.3–43.7	1.02	1.01	1.04	0.0092				
43.8–69.1	0.88	0.86	0.89	<0.0001				
69.2–100	0.66	0.65	0.67	<0.0001				

*Population density per square mile of land area, 2010* ^*∗*^								
0–45	1.00				1.00			
46–114	0.83	0.82	0.84	<0.0001	0.87	0.86	0.88	<0.0001
115–379	0.71	0.70	0.72	<0.0001	0.82	0.81	0.84	<0.0001
380–69468	0.59	0.58	0.60	<0.0001	0.80	0.79	0.81	<0.0001

*% obese, age-adjusted, 2013*								
11.8–28.0	1.00				1.00			
28.0–30.9	1.26	1.25	1.27	<0.0001	1.10	1.09	1.11	<0.0001
31.0–33.7	1.40	1.39	1.41	<0.0001	1.13	1.12	1.14	<0.0001
33.8–47.9	1.60	1.59	1.62	<0.0001	1.18	1.16	1.19	<0.0001

*% leisure-time physical inactivity*, *age-adjusted*, *2013*								
9.7–21.4	1.00							
21.4–24.4	1.18	1.17	1.19	<0.0001				
24.5–28.0	1.29	1.29	1.30	<0.0001				
28.0–39.8	1.60	1.59	1.62	<0.0001				

^*∗*^Altitude and population density groupings represent 50%, 25%, 15%, and 10% of the counties.

**Table 5:** Relative mortality rates of death from suicide and self-inflicted injury according to selected environmental, demographic, and lifestyle variables in the contiguous United States, 2011–15.

Quartile groupings	Adjusted for age, sex, and race				Adjusted for age, sex, race, and the other variables with results listed			
	Relative rate	95% LCL	95% UCL	Pr > ChiSq	Relative rate	95% LCL	95% UCL	Pr > ChiSq
*Average daily sunlight (kJ/m* ^*2*^)								
<15050	1.00							
15050–16105	0.96	0.94	0.97	<0.0001				
16106–17720	1.12	1.11	1.14	<0.0001				
≥17721	1.04	1.03	1.05	<0.0001				

*Altitude (m)* ^*∗*^								
<268	1.00				1.00			
269–476	1.08	1.07	1.10	<0.0001	1.09	1.07	1.11	<0.0001
477–1040	0.99	0.97	1.01	0.1779	1.07	1.05	1.10	<0.0001
≥1041	1.52	1.50	1.55	<0.0001	1.65	1.61	1.69	<0.0001

*Average maximum daily temperature (F)*								
<58.1					1.00			
58.1–64.7	0.89	0.88	0.91	<0.0001	0.94	0.92	0.95	<0.0001
64.8–73.0	0.97	0.96	0.99	0.0008	1.08	1.07	1.10	<0.0001
≥73.1	1.07	1.06	1.09	<0.0001	1.19	1.17	1.21	<0.0001

*Average fine particulate matter (µg/m* ^*3*^)								
<10.5	1.00							
10.5–11.8	0.91	0.90	0.92	<0.0001				
11.9–12.8	1.10	1.08	1.11	<0.0001				
≥12.9	1.04	1.02	1.05	<0.0001				

*Average daily precipitation (mm)*								
<2.08	1.00				1.00			
2.08–2.97	1.02	1.01	1.04	0.0023	1.08	1.06	1.10	<0.0001
2.98–3.37	0.99	0.98	1.01	0.3097	1.09	1.07	1.12	<0.0001
≥3.38	1.00	0.99	1.01	0.9954	1.12	1.10	1.14	<0.0001

*% in poverty, 2013*								
3.0–12.5	1.00				1.00			
12.6–16.3	1.18	1.17	1.20	<0.0001	1.01	0.99	1.02	0.4304
16.4–20.8	1.10	1.09	1.12	<0.0001	0.92	0.91	0.94	<0.0001
20.9–55.1	1.11	1.09	1.14	<0.0001	0.88	0.86	0.90	<0.0001

*% adult smokers*								
9.3–23.6	1.00				1.00			
23.6–26.1	1.24	1.22	1.26	<0.0001	1.19	1.17	1.21	<0.0001
26.2–28.5	1.41	1.39	1.43	<0.0001	1.27	1.25	1.30	<0.0001
28.6–40.3	1.57	1.55	1.60	<0.0001	1.44	1.41	1.47	<0.0001

*% urban, 2010 census*								
0–17.3	1.00							
17.3–43.7	1.17	1.12	1.23	<0.0001				
43.8–69.1	1.01	0.97	1.06	0.5328				
69.2–100	0.75	0.72	0.78	<0.0001				

*Population density per square mile of land area, 2010* ^*∗*^								
0–45	1.00				1.00			
46–114	0.75	0.73	0.78	<0.0001	0.83	0.81	0.86	<0.0001
115–379	0.66	0.64	0.68	<0.0001	0.76	0.74	0.78	<0.0001
380–69468	0.52	0.50	0.53	<0.0001	0.69	0.67	0.71	<0.0001

*% obese, age-adjusted, 2013*								
11.8–28.0	1.00				1.00			
28.0–30.9	1.20	1.18	1.21	<0.0001	1.08	1.07	1.10	<0.0001
31.0–33.7	1.32	1.30	1.34	<0.0001	1.11	1.09	1.13	<0.0001
33.8–47.9	1.44	1.41	1.47	<0.0001	1.12	1.09	1.15	<0.0001

*% leisure-time physical inactivity*, *age-adjusted*, *2013*								
9.7–21.4	1.00							
21.4–24.4	1.08	1.07	1.09	<0.0001				
24.5–28.0	1.16	1.14	1.18	<0.0001				
28.0–39.8	1.39	1.36	1.42	<0.0001				

^*∗*^Altitude and population density groupings represent 50%, 25%, 15%, and 10% of the counties.

## Data Availability

The data used in this study are in the public domain, with references to these data provided in the paper.

## Abbreviations

FIPS: Federal Information Processing Standard
ICD: International Classification of Diseases
kJ/m^2^: Kilojoule/square meter
m: Meter
mm: Millimeters
F: Fahrenheit
*µ*g/m³: Microgram per cubic meter.
